# STARTER Checklist for Antimalarial Therapeutic Efficacy Reporting

**DOI:** 10.4269/ajtmh.22-0224

**Published:** 2022-06-13

**Authors:** Mateusz Plucinski, Elizabeth A. Ashley, Quique Bassat, Meera Venkatesan, Philip J. Rosenthal, Eric S. Halsey

**Affiliations:** ^1^ U.S. President’s Malaria Initiative, Malaria Branch, US Centers for Disease Control and Prevention, Atlanta, Georgia;; ^2^ Lao-Oxford-Mahosot Hospital-Wellcome Trust Research Unit, Vientiane, Laos;; ^3^ Centre for Tropical Medicine and Global Health, Nuffield Department of Medicine, University of Oxford, Oxford, United Kingdom;; ^4^ ISGlobal, Hospital Clínic - Universitat de Barcelona, Barcelona, Spain;; ^5^ Centro de Investigação em Saúde de Manhiça (CISM), Maputo, Mozambique;; ^6^ ICREA, Pg. Lluís Companys 23, 08010 Barcelona, Spain;; ^7^ Pediatrics Department, Hospital Sant Joan de Déu, Universitat de Barcelona, Esplugues, Barcelona, Spain;; ^8^ Consorcio de Investigación Biomédica en Red de Epidemiología y Salud Pública (CIBERESP), Madrid, Spain;; ^9^ U.S. President’s Malaria Initiative, United States Agency for International Development, Washington, District of Columbia;; ^10^ University of California San Francisco, San Francisco, California

Efficacious antimalarials are a cornerstone of the global effort to control and eliminate malaria. However, the spread of drug resistance threatens gains achieved over the early years of this century. Of particular concern is widespread artemisinin resistance in Southeast Asia and its recent emergence in Africa, threatening the efficacy of artemisinin-based combination therapies that currently offer our best treatments for malaria.^1^ Prompt identification of the emergence and spread of antimalarial drug resistance is crucial to guarantee effective case management. At a country level, efficacy data are used by ministries of health and their partners to determine the national treatment guidelines. Because parasites do not respect political and administrative boundaries, coordination of surveillance, and control strategies at the regional and global level is necessary to guide larger containment strategies.

**Table 1:** Standardized Antimalarial Therapeutic Efficacy Reporting (STARTER): Essential items to be included in reports of therapeutic efficacy of antimalarials for uncomplicated malaria

	Item No.	Recommendation	Section
**Introduction**			
	1	(a) Describe current policy for the treatment of malaria	
**Study design and data collection methods**
Summary	2	(a) Provide dates and location(s) of study. For locations, include details at the district and city/village level, if available	
		(b) Specify target sample size and power calculation	
		(c) Define arms by site and drug	
		(d) Describe any randomization or blinding procedures	
Antimalarial studied and dosing specifics	3	(a) Specify antimalarial manufacturer	
		(b) Describe source of medicine and/or quality control measures	
		(c) Provide age or weight bands used for dosing	
		(d) State whether doses were given with or without food	
		(e) State timing of doses and whether all doses (or which doses) were directly observed	
Inclusion and exclusion criteria	4	(a) Provide age range	
		(b) Specify how fever (or history of fever) was measured and defined	
		(c) Present parasite density range for inclusion (if any)	
		(d) Specify minimum acceptable hemoglobin value (if any)	
Patient follow-up	5	(a) List days participants were followed up and approximate time windows	
		(b) Specify treatment of patients in the case of early or late treatment failure	
		(c) Describe clinical and laboratory assessments performed at each follow-up visit	
Outcome definition	6	(a) Define early and late treatment failure	
		(b) Define adequate clinical parasitological response	
		(c) Specify primary efficacy indicator and how it was calculated	
		(d) State how new infections, loss to follow-up, protocol violation, and indeterminate results were figured in primary efficacy calculations (e.g., censored, excluded)	
**Laboratory methods**			
Microscopy	7	(a) State how slides were prepared	
		(b) State how many microscopists read each slide	
		(c) State how discrepancies were defined and resolved	
		(d) State how parasite density was calculated	
Molecular correction (recrudescence vs. new infection)	8	(a) Specify markers used for genotyping	
		(b) State whether all markers were assessed for all samples	
		(c) Describe criteria used to determine new infection vs. recrudescence, including both the definition of a match at each marker and the overall definition of recrudescence considering all markers	
		*For fragment-length polymorphic markers *(e.g., msp1, msp2, *microsatellites*)	
		(d) Specify range of fragment size differences that qualified as a match for each marker	
		(e) Provide cut-off settings for PCR artefacts and stutter peaks	
		(f) State how fragment lengths were measured (e.g., capillary electrophoresis or gel)	
		*For non-fragment-length polymorphic markers (e.g., SNP-barcodes, amplicon sequencing)*	
		(g) Describe sequencing methodology	
		(h) Provide sequencing depth and cut-offs	
		(i) Cite bioinformatics software and workflow	
**Data and results**			
Patients reaching study outcomes	9	(a) Provide number of participants enrolled, lost to follow-up, withdrawn, and excluded	
		(b) State reasons for exclusion	
Participant composition by arm	10	Describe age, sex, initial parasite density, and initial hemoglobin	
Outcome by arm	11	(a) Provide % slide positivity amongst patients seen on Day 3 (with Day 0 defined as first day of treatment)	
		(b) List number of late treatment failures classified as new infections, recrudescences, or indeterminate	
		(c) List number of participants with adequate clinical and parasitological response	
		(d) Report day 28 results for arms with follow up ≥ 28 days	
		(e) Report day 42 results for all arms with follow up ≥ 42 days	
		(f) Provide Kaplan–Meier estimates of efficacy, where new infections and cases with loss to follow up are censored	
		(g) Provide estimates and confidence intervals of both uncorrected and PCR-corrected results	
		(h) Disaggregate all outcomes by study arm (site, drug, and species)	
		(i) Only calculate *P* values if study was specifically designed and powered to detect a difference between arms	
Genotyping data	12	Provide table or supplementary table of paired full genotyping data (observed alleles at each locus) and classification for each late treatment failure.	

Note: Please refer to WHO guidance for antimalarial efficacy monitoring, molecular techniques, and distinguishing reinfection from recrudescence after therapy.^2^^–^^4^ Commonly found errors in therapeutic efficacy reports have been characterized recently.^1^^,^^5^

Since the 1960s, the foundation of antimalarial drug efficacy monitoring has been the use of therapeutic efficacy studies that monitor parasitological and clinical response in patients treated for malaria. Standardized methods for performance and analysis of these studies are codified in World Health Organization (WHO) guidance documents.^2^^–^^4^ Despite the standardized guidance, recent investigation has identified frequent nonadherence to the WHO guidelines.^1^^,^^5^ Notably, deviations from the standard of practice methodology are common, particularly related to analysis of genotyping data and definition of primary outcome indicators, and critical methodological details are often omitted from publications reporting efficacy data. As a consequence, there is the risk that reported efficacy could either be underestimated or overestimated, with readers not able to determine both the scope and the direction of the under- or overestimates. Such a loss in accuracy can obstruct the global effort to prevent and contain antimalarial drug resistance.

Incorporating comments from the WHO Global Malaria Program, the Malaria Branch of the Centers for Disease Control and Prevention, the U.S. President’s Malaria Initiative, and the editors-in-chief and antimalarial efficacy section leads from the *American Journal of Tropical Medicine and Hygiene* and *Malaria Journal*, we have developed the Standardized Antimalarial Therapeutic Efficacy Reporting (STARTER) Checklist, which lays out best practices for reporting the results of antimalarial efficacy studies. Similar to other reporting checklists, it has been registered and is available at the EQUATOR Network repository (Table 1).^6^

We emphasize that checklists are not replacements for peer-review,^7^ but rather tools to promote uniformity in reporting. Filling out the checklist does not substitute for careful adherence to the global WHO standards for efficacy trials. Efficacy trial investigators and sponsors that follow WHO’s guidance and verify their adherence at the protocol development, implementation, analysis, and reporting stages will likely find the STARTER checklist facilitates their manuscript development.

## References

[1] World Health Organization , 2020. *Report on Antimalarial Drug Efficacy, Resistance and Response: 10 Years of Surveillance (2010–2019)*. Geneva, Switzerland: World Health Organization.

[2] World Health Organization , 2009. *Methods for Surveillance of Antimalarial Drug Efficacy*. Geneva, Switzerland: World Health Organization.

[3] World Health Organization , 2007. *Methods and Techniques for Clinical Trials on Antimalarial Drug Efficacy: Genotyping to Identify Parasite Populations*. Geneva, Switzerland: World Health Organization.

[4] World Health Organization , *Informal Consultation on Methodology to Distinguish Reinfection from Recrudescence in High Malaria Transmission Areas*. Available at: https://www.who.int/publications-detail-redirect/9789240038363.

[5] Plucinski M Hastings I Moriarty LF Venkatesan M Felger I Halsey ES, 2021. Variation in calculating and reporting antimalarial efficacy against *Plasmodium falciparum* in sub-Saharan Africa: a systematic review of published reports. Am J Trop Med Hyg 104: 1820–1829. 33724925 10.4269/ajtmh.20-1481 PMC8103451

[6] *The EQUATOR Network | Enhancing the QUAlity and Transparency Of Health Research*. Available at: https://www.equator-network.org/.

[7] Hirji KF, 2009. No short-cut in assessing trial quality: a case study. Trials 10: 1. 19128475 10.1186/1745-6215-10-1 PMC2636799

